# Computational prediction of associations between long non-coding RNAs and proteins

**DOI:** 10.1186/1471-2164-14-651

**Published:** 2013-09-24

**Authors:** Qiongshi Lu, Sijin Ren, Ming Lu, Yong Zhang, Dahai Zhu, Xuegong Zhang, Tingting Li

**Affiliations:** 1 Department of Biomedical Informatics, School of Basic Medical Sciences, Peking University Health Science Center, Beijing 100191, China; 2 MOE Key Laboratory of Bioinformatics and Bioinformatics Division, TNLIST/Department of Automation, Tsinghua University, Beijing 100084, China; 3 Institute of Systems Biomedicine, School of Basic Medical Sciences, Peking University Health Science Center, Beijing 100191, China; 4 National Laboratory of Medical Molecular Biology, Institute of Basic Medical Sciences, Chinese Academy of Medical Sciences, School of Basic Medicine, Peking Union Medical College, Beijing, China; 5 Current address: Department of Biostatistics, Yale University, New Haven, CT 06511, USA

**Keywords:** Long non-coding RNA, RNA–protein interaction, Computation

## Abstract

**Background:**

Though most of the transcripts are long non-coding RNAs (lncRNAs), little is known about their functions. lncRNAs usually function through interactions with proteins, which implies the importance of identifying the binding proteins of lncRNAs in understanding the molecular mechanisms underlying the functions of lncRNAs. Only a few approaches are available for predicting interactions between lncRNAs and proteins. In this study, we introduce a new method lncPro.

**Results:**

By encoding RNA and protein sequences into numeric vectors, we used matrix multiplication to score each RNA–protein pair. This score can be used to measure the interactions between an RNA–protein pair. This method effectively discriminates interacting and non-interacting RNA–protein pairs and predicts RNA–protein interactions within a given complex. Applying this method on all human proteins, we found that the long non-coding RNAs we collected tend to interact with nuclear proteins and RNA-binding proteins.

**Conclusions:**

Compared with the existing approaches, our method shortens the time for training matrix and obtains optimal results based on the model being used. The ability of predicting the associations between lncRNAs and proteins has also been enhanced. Our method provides an idea on how to integrate different information into the prediction process.

## Background

Recent studies show that only a small part of the human transcriptome is involved in the protein-coding process [1]. Long non-coding RNAs (lncRNAs) comprise the majority of transcripts; however, little is known about the function of lncRNAs [2]. The GENCODE project discovered more than 14,000 lncRNA transcripts from approximately 9,000 gene loci [3]. The lncRNA database collected a few hundred of high-confidence, experimentally validated lncRNAs [4]. For example, the Xist RNA of humans is a large RNA sequence (19 kb) that has remained untranslated [5]. Xist RNA has been proven to have an important function in regulating X-inactivation [6,7]. Although an increasing list of evidence demonstrates that lncRNAs may be involved in multiple biological processes, including epigenetic regulation, chromatin remodeling, and cell proliferation and differentiation, the molecular mechanisms underlying the functions of lncRNAs still remain largely elusive.

In general, lncRNAs function with their binding proteins. Thus, in order to understand the molecular mechanisms underlying the functions of lncRNAs, the binding proteins of lncRNAs need to be identified. Several experimental approaches such as RNA immunoprecipitation followed by mass spectrometry analysis have been developed to identify the lncRNA binding proteins. Recently, Bellucci et al. [8] developed a computational method CatRAPID for predicting RNA–protein interactions. They further used the method on the predictions of protein interactions in the Xist network [9]. The method involves encoding protein-RNA pairs into feature vectors, identifying a matrix, and calculating the interaction score through matrix computation. Their results showed that the method is powerful and may be used to predict RNA–protein interactions from sequences.

This study aims to explore a new method, lncPro, for predicting lncRNA–protein interactions. lncPro yields a score using amino acid and nucleotide sequences. This score can be used to measure the interaction between a pair of lncRNA and protein. Fisher’s linear discriminant method was used to compute the matrix directly. Based on the mathematical model being used, the result was found to be theoretically optimal in the sense of discriminant. Applying lncPro on all human proteins, we found that the long non-coding RNAs we collected tend to interact with nuclear proteins and RNA-binding proteins. A convenient online server (http://cmbi.bjmu.edu.cn/lncpro) has been developed for lncPro.

## Methods

### Data sorting

A training set containing many pairs of proteins and RNAs is needed. Information on the interactivity of each pair is also required.

Complexes were downloaded from the Protein Data Bank (PDB) database (http://www.pdb.org). RNA–protein pairs were extracted from these complexes. Although the number of pairs is large, many sequences from the same complex are identical according to PDB. These repeated pairs were deleted. Take complex 2FTC as an example. 2FTC has 18 chains, but chain E and chain F are identical. Thus, we included 17 chains (one RNA sequence and 16 protein sequences) in the training set. Following this procedure, a training set containing 1761 pairs was obtained from 44 complexes (Additional file 1: Table S1). Among the RNAs in the training set, some sequences are noticeably shorter than the long non-coding RNAs (more than 200 bp) we wish to study. When deleting all the sequences shorter than 200 bp, this led to a small training set, therefore sequences longer than 100 bp were kept in the training set. This selection was an approximation and not all the RNA sequences contained in our training set are long non-coding RNAs. Following the procedure above, a training set containing 726 pairs was obtained. These remaining pairs came from 18 complexes (Additional file 2: Table S2). The corresponding complexes and organisms are listed in Table 1.

**Table 1:** Complexes used in the training set

**Organism**	**Complex ID (PDB database)**
*Haloarcula marismortui*	1FFK, 1JJ2
*Thermus thermophiles*	1GIY, 3HUW, 3I8I
*Deinococcus radiodurans*	1J5A, 2ZJP
*Escherichia coli*	1P85, 2GYA
*Bos Taurus*	2FTC
*Mus musculus*	2R8S
*Neurospora crassa*	2RKJ
*Canis lupus familiaris*	2ZKQ, 2ZKR
*Spinacia oleracea*	3BBN, 3BBO
*Homo sapiens*	3CW1
*Thermomyces lanuginosus*	3JYV

After collecting RNA–protein pairs, a criterion is needed to determine whether a pair is interactive or non-interactive. The “least atom distance” was used as the criterion [10]: assume that R is an RNA molecule and P is a protein molecule. If there exists an atom r of R and an atom p of P such that the distance between r and p is less than 5 Å, the pair (R and P) is considered to be interactive. Otherwise, the pair is non-interactive. The distance cutoff 5 Å was borrowed from the PRIDB database [10]. RNA–protein pairs in the training set can now be classified based on interactivity. Each pair in the training set was checked, yielding 355 interactive pairs and 371 non-interactive pairs. Since some sequences in this dataset are similar, this set will be called the redundant training set in the following sections. If these highly similar sequences were assigned to the training set and test set respectively, the evaluation of the performance would be biased. The CD-HIT tool (http://weizhong-lab.ucsd.edu/cdhit_suite/cgi-bin) was used to compute sequence similarity for both protein and RNA sequences. Pairs that share both the protein sequence and the RNA sequence are considered to be similar, and thus removed from the training set. The similarity cutoff was set at 90% for both protein and RNA. After redundancy removal, a training set containing 649 protein-RNA pairs, including 322 interactive pairs and 327 non-interactive pairs, was obtained. This set will be called the non-redundant training set.

### Feature vector encoding

**Figure 1:**
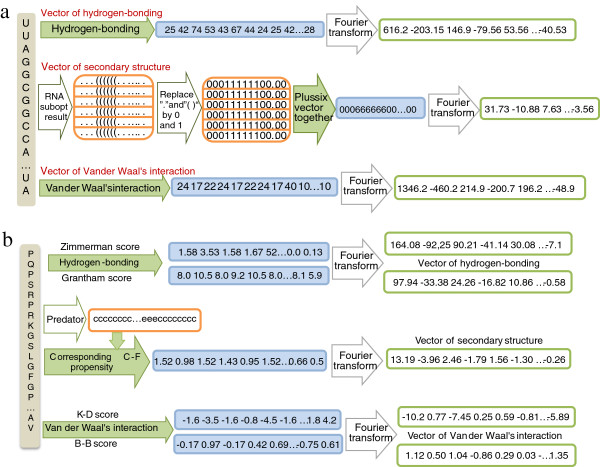
**Procedure of encoding the RNA sequences and the amino acid sequences into feature vectors. a)** Procedure of encoding the RNA sequences into feature vectors. For the secondary structure, RNAsubopt was used to obtain the top six possible secondary structures with the lowest free energy. The dots and brackets were then replaced by 0 s and 1 s, respectively. The six vectors were added, and the secondary structure feature vector was obtained. For Van der Waal’s interaction and hydrogen bonding, each base was replaced by numbers representing the propensities. Finally, all three feature vectors were transformed by the Fourier series, and the first 10 terms of Fourier series were used as the new feature vector. **b)** Procedure of encoding the amino acid sequences into feature vectors. For the feature vector of the secondary structure, the corresponding Chou-Fasman propensities were used to encode each amino acid according to the secondary structure predicted by Predator. For the feature vectors of hydrogen bonding, each amino acid was replaced by Grantham’s and Zimmerman’s scores, respectively. Kyte-Doolittle and Bull-Breese scores were used for Van der Waals’ interaction, respectively. For all five feature vectors, the first 10 terms of the Fourier series were used as new feature vectors.

This section will focus on encoding sequences into numerical feature vectors. Information from the secondary structure, the hydrogen-bonding propensities, and the Van der Waals’ propensities was used. The encoding procedure is presented in Figure 1. Details for each vector are described below.

### Secondary structure propensities

RNAsubopt from the Vienna Package [11] was used to predict the secondary structure of RNAs. RNAsubopt provides *n* possible forms of secondary structure with the lowest free energy. Different selections of *n* may lead to differences in performance. The Discriminative Power (Details of discriminative power can be found in the “Measuring the performance of the method” section) is used as an indicator of the performance. The Discriminative Power does not change significantly when *n* is set at values of 5, 6, 7, or 8 (4-fold cross validation gave discriminative power values of 90.5, 90.3, 91.2, and 91.6, respectively). Since a larger *n* value brings more computation, *n* was set at six. When an RNA sequence was given, six results were obtained in the form of dots and brackets. Each bracket was replaced with “1”, and each dot was replaced with “0”. Thereafter, six binary sequences were added to obtain a new feature vector with integers between zero and six.

Predator [12] software was used to predict the secondary structure of proteins. Chou-Fasman propensities were used to encode each amino acid [13]. By replacing each amino acid in the sequence with the corresponding Chou-Fasman propensity, the sequence was transformed into a numerical feature vector.

### Hydrogen-bonding propensities and Van der Waals’ propensities

Purine and pyrimidine contact information from a set of 41 RNA–protein complexes [14] was used to encode the RNA numerical feature vectors for hydrogen bonding and Van der Waals’ interaction. The RNA feature vectors showed the propensities of the atoms to form hydrogen bonds and Van der Waals’ interaction. The performance evaluation may be biased if the set of 41 RNA–protein complexes for purine and pyrimidine contact information have significant overlaps with our training set. Among these 41 complexes, only one complex (1JJ2) is shared with our training set and 12 RNA-protein pairs are involved. We further used the CD-HIT tool to check whether there are significant similarities for the other sequences. When the cutoff was set at 0.9 for both RNA and protein, only three RNA-protein pairs are shared between our training set and those 41 complexes. Therefore, these 41 RNA–protein complexes for purine and pyrimidine contact information do not have significant overlap with the training set.

For the proteins, hydrogen bonding feature vectors were encoded using Grantham’s propensities [15] and Zimmerman’s propensities [16]. Feature vectors of Van der Waals’ interaction were encoded using Kyte-Doolittle [17] and Bull-Breese propensities [18]. Together with the secondary structure feature vector, each protein sequence was encoded into five numerical feature vectors.

### Transformation of the dimension

Each protein sequence and each RNA sequence were transformed into five and three numerical feature vectors, respectively. However, these vectors cannot be used for direct computations because the dimension of each vector depends on the length of the corresponding RNA or protein sequence, which makes it impossible to find a fixed matrix **M** to conduct the computation. Therefore, the vectors need to be transformed in order to unify the dimension.

The Fourier series was used to solve the problem. The formula is presented as follows:

(1) $$\begin{array}{l} X_{k}^{'}=\sqrt{\frac{2}{L}}\overset{L}{\underset{n=0}{\sum }}X_{n}\cos\left[\frac{\pi }{L}\left(n+\frac{1}{2}\right)\left(k+\frac{1}{2}\right)\right]\;,k\\ \;=0,1,\ldots ,9 \end{array}$$

Where *L* is the length of the original feature vector.

Here, the first 10 terms of the Fourier series were used as the new numerical feature vector. Dimension 10 was selected because the results did not improve significantly with a higher dimension and the computation is faster when the dimension is set lower. When the dimension was set at 10, 15, and 20, we acquired Discriminative Power (Details of discriminative power can be found in the “Measuring the performance of the method” section) values 90.3%, 89.8%, and 91.9% respectively. After transforming each RNA–protein pair into eight 10-dimensional numerical feature vectors (three for RNA and five for protein), the feature vector encoding process was completed.

### Finding the matrix

For each pair of feature vectors **r** and **p** (representing the RNA feature vector and the protein feature vector, respectively), we want to train a matrix **M** and use the score <**p**|**M**|**r** > to measure the interaction between **r** and **p**. **M** will be a 100-D matrix because the dimension of vectors was set at 10. If we unsystematically search the matrix in the 100-D Euclidean space, the efficiency and accuracy would be low. The efficiency and accuracy will be further degraded when a higher dimension is used.

Let us analyze the expansion of <**p**|**M**|**r**>. Without loss of generality, the situation of dimension two is used to clarify the idea:

Assuming that **p** = (*p*_*1*_*, p*_*2*_), **r** = (*r*_*1*_*, r*_*2*_)^T^,

$M=\left(\begin{array}{cc} M_{1} & M_{2}\\ M_{3} & M_{4} \end{array}\right)$, then

(2) $$\begin{array}{l} <p|M|r>=\left(p_{1}\;p_{2}\right)\left(\begin{array}{cc} M_{1} & M_{2}\\ M_{3} & M_{4} \end{array}\right)\;\left(\begin{array}{c} r_{1}\\ r_{2} \end{array}\right)\\ \;=p_{1}M_{1}r_{1}+p_{1}M_{2}r_{2}+p_{2}M_{3}r_{1}\\ \;+p_{2}M_{4}r_{2} \end{array}$$

Equivalently, we can write <**p**|**M**|**r** > as follows:

(3) $$\begin{array}{l} <p|M|r>=M_{1}p_{1}r_{1}+M_{2}p_{1}r_{2}+M_{3}p_{2}r_{1}\\ \;+M_{4}p_{2}r_{2}=\mathrm{kx} \end{array}$$

Here, **k** = (*M*_1_, *M*_2_, *M*_3_, *M*_4_), **x** = (*p*_1_*r*_1_, *p*_1_*r*_2_, *p*_2_*r*_1_, *p*_2_*r*_2_)^T^.

When given **p** and **r**, then **x** will be fixed. Therefore, the idea of finding the matrix **M** is equivalent to finding the vector **k**. The score <**p**|**M**|**r** > is actually the inner product of vectors **x** and **k**. This inner product score is expected to discriminate the data into two groups. Thus, according to the theory of Fisher’s linear discriminant method, the best vector **k** is actually the direction **k** to optimize the Fisher criterion function:

(4) $$J\left(k\right)=\frac{{\left(m_{1}‒m_{2}\right)}^{2}}{s_{1}^{2}+s_{2}^{2}}$$

Here, *m*_*i*_ represents the mean of each category. $s_{i}^{2}=\sum_{x\in C_{i}}{\left(x‒m_{i}\right)}^{2}$. The subscript denotes two different classes.

In the ten-dimensional case, the whole procedure is similar. We can obtain the following:

(5) <p|M|r>=kx

Where **k** = (*M*_1_, *M*_2_, …, *M*_100_) and **x** = (*p*_1_*r*_1_, *p*_1_*r*_2_, …, *p*_10_*r*_10_)^T^; both are 100-dimensional vectors. After transforming each pair of **r** and **p** in the training set into (*p*_1_*r*_1_, *p*_1_*r*_2_, …, *p*_10_*r*_10_)^T^, Fisher’s linear discriminant method was applied to these 100-dimensional vectors. Subsequently, the optimal direction **k** was computed directly.

Thus, the problem of finding a matrix **M** in the 100-dimensional Euclidean space was transformed into another equivalent problem of finding a vector **k**. Using Fisher’s linear discriminant method, **k** was computed directly from known data, thereby simplifying the process. Based on the mathematical model being used, the obtained result was found to be theoretically optimal.

### Combining the feature vectors

Information on the secondary structure, hydrogen bonding propensities, and Van der Waals’ interaction has to be integrated. We can simply add the different feature vectors into a new vector that contains information from different aspects, |**p** > = |**p**_**1**_ > + |**p**_**2**_ > + |**p**_**3**_ > + |**p**_**4**_ > + |**p**_**5**_ >, |**r** > = |**r**_**1**_ > + |**r**_**2**_ > + |**r**_**3**_ >. Thereafter, the computation can be performed directly with this new feature vector.

(6) $$\begin{array}{l} <p|M|r>=\left(<p_{1}|+<p_{2}|+<p_{3}|+<p_{4}|+\right)\\ \;\left(<p_{5}|\right)M\left(|r_{1}>+|r_{2}>+|r_{3}>\right) \end{array}$$

However, Equation (6) will lead to cross terms such as <**p**_**1**_|**M**|**r**_**2**_ >. Assuming that **p**_**1**_ indicates the protein secondary structure information and that **r**_**2**_ indicates the RNA hydrogen bonding information, then the computation of <**p**_**1**_|**M**|**r**_**2**_ > is nonsensical because combining different kinds of information is theoretically meaningless. Thus, we selected another combining method. We computed five scores using each feature vector pairs, respectively, which included: the protein and RNA secondary structures, protein Grantham’s propensities and RNA hydrogen bonding, protein Zimmerman’s propensities and RNA hydrogen bonding, protein Kyte-Doolittle propensities and RNA Van der Waals’ interaction, and protein Bull-Breese propensities and RNA Van der Waals’ interaction. Here the Grantham and Zimmerman scores are both characterizing the protein hydrogen bonds, the Kyte-Doolittle and Bull-Breese scores are both characterizing the protein Van der Waals’ interaction.

The five scores must be combined to generate a final score. Given that the encoding methods of feature vectors are different, their magnitudes are also different. As a result, the five scores cannot be combined directly. Thus, we transform each score into open interval (0,100). This is realized by a 1–1 map from the real line to (0,100):

(7) $$Y=\frac{100}{\pi }\text{arctan}\;\left(\frac{2\left(X‒c\right)}{c_{1}‒c_{2}}\right)+50$$

Where *X* is the raw score, *Y* is the transformed score, *c*_*1*_ = **k** * **m**_**1**_, *c*_*2*_*=***k** * **m**_**2**_, and *c = (c*_*1*_*+ c*_*2*_*)*/2 is the mean of *c*_*1*_ and *c*_*2*_. In *c*_*1*_*=*k*m_**1**_, and *c*_*2*_*=*k*m_**2**_, **m**_**1**_ and **m**_**2**_ are the mean vectors of positive and negative sets, respectively. *c = (c*_*1*_*+ c*_*2*_*)*/2 can be considered as the separate point of interactive pairs and non-interactive pairs. If *X*> >*c*, then *Y* is near 100. If *X* < <*c*, then *Y* is near zero. If *X* = *c*, then *Y* = 50. Thus, the cutoff will be decided at 50, naturally.

According to this formula, we can transform all five scores into a scale ranging from 0 to 100. We considered two options to combine the five scores. The first is the arithmetic mean of the five scores. The second is to set weights at 1/3, 1/6, 1/6, 1/6, and 1/6, respectively, considering the Grantham and Zimmerman scores are both characterizing hydrogen bonds and the Kyte-Doolittle and Bull-Breese scores are both characterizing the Van der Waals’ interaction. We observed the prediction of different cases. The former method is more accurate than the latter. Therefore, the arithmetic mean of the five scores was used as the final score.

### Measuring the performance of the method

The definition of Discriminative Power (DP) was adopted from the study of Bellucci et al. [8].

(8) $$\mathrm{DP}=\frac{\sum_{i}\sum_{n}\theta \left(\pi_{i}‒\pi_{n}\right)}{\sum_{i}\sum_{n}\theta \left(\pi_{i}‒\pi_{n}\right)+\sum_{i}\sum_{n}\theta \left(\pi_{n}‒\pi_{i}\right)}$$

Where *π*_*i*_ and *π*_*n*_ represent the scores of interactive and non-interactive RNA–protein pairs, respectively. The function *θ* is defined as follows:

(9) $$\theta \left(x\right)=\left\{\begin{array}{c} 1,\;\mathrm{if}\;x\geq 0\\ 0,\;\mathrm{if}\;x<0 \end{array}\right)$$

After all pairs were reordered with the score provided by our method, DP = 1 if all the interactive pairs are ordered before the non-interactive ones. If all the non-interactive pairs are ordered before interactive ones, then DP = 0. Thus, DP can be used to determine whether the method could discriminate the positive and negative sets well.

Furthermore, each pair was given a score between 0 and 100 using the method above. The cutoff is naturally decided as 50 according to the formula we used. Thus, a pair is interactive if it has a score over 50; otherwise, the pair is non-interactive. Using this cutoff, we can calculate the accuracy of the prediction.

Besides the DP value defined above, Matthews correlation coefficients (MCC) can also be used to measure the quality of classification:

(10) $$\mathrm{MCC}=\frac{\mathrm{TP}\times \mathrm{TN}-\mathrm{FP}\times \mathrm{FN}}{\sqrt{\left(\mathrm{TP}+\mathrm{FP}\right)\left(\mathrm{TP}+\mathrm{FN}\right)\left(\mathrm{TN}+\mathrm{FP}\right)\left(\mathrm{TN}+\mathrm{FN}\right)}}$$

Here, *TP* is the number of true positives; *FP* is the number of false positives; *TN* is the number of true negatives; *FN* is the number of false negatives.

## Results and discussion

First, we used cross-validation to test the classification ability of the method on the training set. Then the ncRNA–Protein Interaction (NPInter) database and several complexes were used to test the ability of the prediction. Finally, we used the method on all human proteins.

### Cross-validation on training set and comparison with CatRAPID

Since the CD-HIT tool has been used to delete similar sequences, a 4-fold cross-validation could be performed on the non-redundant training set. The mean DP was taken as the final result. Our method obtained a DP value 90.3% on the non-redundant set. Since there are some similar pairs in the redundant set, a higher discriminative power value 94.3% was obtained on the redundant set. Compared with the results of CatRAPID (78% on the non-redundant set and 90% on the redundant set), this method showed a better ability to discriminate interactive and non-interactive pairs. The Matthews Correlation Coefficient (MCC) was also computed. We obtained an MCC value of 0.74 on the non-redundant set and a higher MCC value of 0.83 on the redundant set. This is consistent with the previous DP results.

CatRAPID used 7 Å as the cutoff between interacting and non-interacting RNA-protein pairs and RNA sequences shorter than 100 bp were also kept in the training set. To compare with CatRAPID, the method was also tested on the other set when distance cutoff was set at 7 Å and RNA sequences shorter than 100 bp were kept in the training set. A 4 fold cross-validation was performed. We obtained DP values of 90.2% and 91.6% on the non-redundant set and the redundant set, respectively, which were more accurate than CatRAPID. MCC values were 0.77 on the non-redundant set and 0.78 on the redundant set.

Next, we checked if the results were improved when five scores were combined. If the combining results were less accurate than the prediction using any single score, then the combination of different information would be meaningless. We considered the five scores separately. The DP for each score is presented in Table 2. The DPs of different scores were not at the same level. However, the combining result was more accurate than the result of each single group. This proved that our method of combining was effective. The same process was repeated on the redundant set. The DP value obtained after combination was still the highest (Table 3). It could be noticed that the DP value is lower than the values of other groups when considering the B-B score alone. When including other 4 groups in the model except the B-B score, the DP value was 89.1% on the non-redundant set and 93.2% on the redundant set. Thus, the B-B score was kept in the model. These results showed that the five groups of vectors do contain different information that is related to RNA-protein interaction. So the classification is more accurate when more groups are used together. Even though K-D and B-B scores are both using information of Van der Waal’s propensities, Grantham and Zimmerman scores are both using information of hydrogen bonding, the ways these vectors were coded are very different.

**Table 2:** Discriminative power for each score (non-redundant set)

	**Sec-structure**	**Grantham**	**Zimmerman**	**K-D**	**B-B**	**Combination**
DP	88.1%	88.1%	89.1%	87.8%	65.6%	90.3%

**Table 3:** Discriminative power for each score (redundant set)

	**Sec-structure**	**Grantham**	**Zimmerman**	**K-D**	**B-B**	**Combination**
DP	90.0%	92.0%	91.5%	89.7%	73.6%	94.3%

We further checked whether a strict sequence similarity cutoff for the non-redundant training set would influence the prediction performance dramatically. When a strict sequence similarity cutoff was used (0.3 for protein and 0.8 for RNA), 21 pairs were deleted from the non-redundant training set. A DP value of 87.1% was then obtained. The result was still more accurate than CatRAPID (78% on the non-redundant set).

### Testing on NPInter database

In the following section, we used the whole non-redundant training set to train the method, and tested the method’s performance on another database. The NPInter database (http://bioinfo.ibp.ac.cn/NPInter/) contains many ncRNA–protein pairs from different species including Homo sapiens, Mus musculus, Escherichia coli, Drosophila melanogaster and Saccharomyces cerevisiae. These ncRNA–protein pairs can be separated into eight groups [19].

1. ncRNA binds to the protein;

2. ncRNA regulates the mRNA;

3. ncRNA indirectly regulates a gene;

4. ncRNA is regulated by the protein;

5. ncRNA as a factor affects the protein’s function;

6. The protein as a factor affects the ncRNA’s function;

7. Genetic linkages between the ncRNA and the protein;

8. Special linkages between the ncRNA and the protein.

All pairs belong to a positive set (interactive). We considered groups 1, 5, and 6 to have direct evidence of interaction. The rest of the groups were considered to have indirect evidence. Since the RNAsubopt software will only process the first 4095 nucleotides if the input RNA is too long, sequences longer than 4095 were deleted. Finally, 74 pairs with direct evidence of interaction and 41 pairs with indirect evidence were obtained. The CD-HIT tool was used to check if there is significant similarity between the training set and the pairs from NPInter. When both cutoffs were set at 0.9 for protein and RNA sequences, CD-HIT showed that there was no overlapping. Among the 74 pairs with direct evidence, 48 pairs have scores over 50, suggesting that 65% of the interactive pairs were predicted. Among the 41 pairs with indirect evidence, we predicted 27 pairs, which accounted for 66%.

Both pairs with direct and indirect evidence (115 pairs) were used as the positive set. Then we used another 115 pairs randomly from the non-redundant negative training set as the negative set. A DP value of 91.7% was obtained, which was higher than the result of CatRAPID (85%). The MCC value was 0.64, which also indicates acceptable classification ability.

By shuffling pairs in the non-redundant negative training set, we built a randomized set containing 327 pairs. The mean value of predicted interaction scores on this set was 32.71 and the distribution of the predicted interaction scores is displayed in Additional file 3: Figure S1. We further randomly selected 115 of them as the negative set. When this new negative set was used, a DP value of 86.7% was obtained for the positive set, which includes both pairs with direct and indirect evidence (115 pairs). These results confirmed that the classification performance of lncPro is satisfactory.

### Testing on complexes

We also studied the performance of the method on several complexes. First, we took MRP, RNase P, PRC-2, and LSD1/CoREST/REST complex into consideration. The RNA sequences of MRP and RNase P were obtained from the Functional RNA Database (fRNAdb; http://www.ncrna.org/frnadb). The sequence of HOTAIR and MEG3 were downloaded from the National Center for Biotechnology Information database (http://www.ncbi.nlm.nih.gov/). The protein sequences were obtained from the Uniprot database (http://www.uniprot.org/).

The human MRP complex contains one RNA sequence and ten protein sequences: hPop1, hPop5, Rpp14, Rpp20, Rpp21, Rpp25, Rpp29, Rpp30, Rpp38, and Rpp40. RNase P is another complex inside the human body. RNase P shares proteins with the MRP system but has a different RNA sequence. Previous studies focused on MRP and RNase P [20,21]. The results can be summarized as follows. hPop1, Rpp20, Rpp21, Rpp25, Rpp29, and Rpp38 have direct interactions with the corresponding RNA, whereas hPop5, Rpp14, Rpp30, and Rpp40 have relatively weak interactions. We applied the method on these two complexes. The results are shown in Table 4. Considering hPop5, Rpp14, Rpp30, and Rpp40 are non-interactive, we then correctly predicted 70% (7 of 10) of the interactions for the MRP and RNase P complexes.

It is worth noting that most of the complexes (14 out of 18) we used to train the model exist in ribosome, which could potentially lead to a biased prediction model. Therefore, in order to validate the method, lncPro needs to be tested on non-ribosome complexes. The PCR-2 complex contains four protein units: Ezh2, Eed, Suz12, and RBBP4. Their Uniprot IDs are Q15910, O75530, Q15022, and Q09028, respectively. All four proteins were predicted as interactive with HOTAIR (has a score above 50). Ezh2 was predicted to be the main RNA-binding unit. These results are in agreement with known experimental results [22,23]. The interaction scores are listed in Table 5. Besides HOTAIR, it is known that MEG3 also interacts with PCR-2 [24]. lncPro successfully predicted these interactions as well (Table 5). The LSD1/CoREST/REST complex contains three protein units: LSD1, CoREST, and REST. The corresponding Uniprot IDs are O60341, Q9UKL0, and Q13127. All these three proteins are predicted as interactive with HOTAIR (Table 6), among which LSD1 is predicted as the main RNA-binding unit. These predictions are also consistent with known experimental results [25,26]. Therefore, the acceptable prediction performance on non-ribosome complexes proved that our model is valid.

**Table 4:** Interaction scores of MRP and RNase P

**Protein**	**MRP result**	MRP exp*	**RNase P result**	RNase P exp*
hPop1	60.1	+	86.7	+
hPop5	45.2	-	25.2	-
Rpp14	40.9	-	32.9	-
Rpp20	31.2	+	44.3	+
Rpp21	64.7	+	77.6	+
Rpp25	39.8	+	70.0	+
Rpp29	69.6	+	66.8	+
Rpp30	46.5	-	55.7	-
Rpp38	58.0	+	61.4	+
Rpp40	60.7	-	65.6	-

*Known experimental result: “+” indicates interactive, “-” indicates non-interactive.

**Table 5:** Interaction scores of PRC-2 with HOTAIR and MEG3

**Protein**	**HOTAIR**	**MEG3**
Ezh2	93.1	87.4
Eed	63.8	67.6
Suz12	89.7	69.5
RBBP4	59.9	88.8

**Table 6:** Interaction scores of LSD1/CoREST/REST complex and HOTAIR

**Protein**	**Score**
LSD1	90.4
CoREST	73.2
REST	88.8

Next, lncPro was tested on non-ribosome complexes of other species besides human. The MSL complex of Drosophila melanogaster contains two RNA sequences (roX1 and roX2; collected from http://www.noncode.org) and five protein sequences (MSL1, MSL2, MSL3, MLE, and MOF; collected from Uniprot database). It is known that both roX1 and roX2 have interactions with MSL1, MSL2, MSL3, and MLE [27,28]. The prediction scores given by lncPro are presented in Table 7. Since all eight predicted interaction scores between two RNAs and these four proteins are above 50, both roX1 and roX2 are predicted to be interactive with MSL1, MSL2, MSL3, and MLE. This is in agreement with known results. The predicted interaction scores between roX1, roX2 and MOF are also above 50, which suggest that MOF might also have potential interaction with roX1 and roX2.

**Table 7:** Interaction scores with roX1 and roX2

**Protein**	**RoX1 score**	**RoX2 score**
MSL1	75.02	90.76
MSL2	67.39	89.28
MSL3	82.50	66.56
MLE	67.71	57.05
MOF	74.83	59.33

### Score distribution of nuclear proteins and RNA-binding proteins

Most well-studied lncRNAs are located in the nucleus of cells. Recently, non-coding RNAs have been found to be predominantly localized in the nucleus [29]. Therefore, it is natural to obtain higher interaction scores between nuclear proteins and non-coding RNAs. We used our method to see whether the scores of the nuclear proteins are indeed higher than those of other proteins.

**Figure 2:**
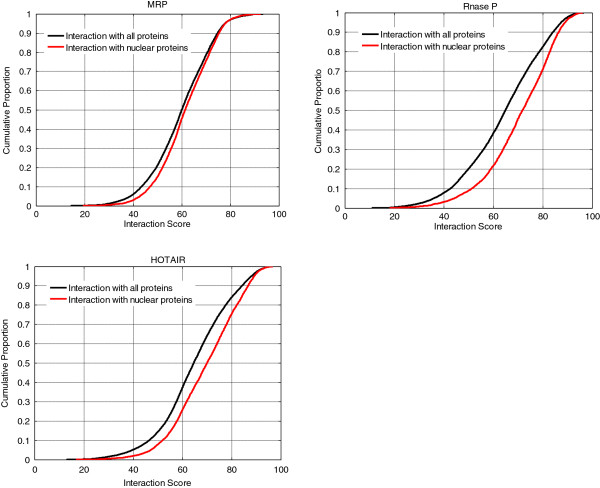
**Cumulative distribution functions (CDF) of different RNAs.** The black curve is the CDF of scores between this RNA and human proteins, and the red curve is the CDF of scores between this RNA and human nuclear proteins. The x-axis indicates the score obtained by our method; the Y-axis indicates proportion.

Protein sequences of Homo sapiens were downloaded from the Swiss-Prot database (20224 protein sequences). Among these 20224 protein sequences, 5575 were annotated as the nuclear protein according to the Cellular Component of Gene Ontology (GO:0005634, nucleus, downloaded from BioMart). The ID lists of 20224 overall proteins and 5575 nuclear proteins were presented in Additional file 4: Table S3. For each given lncRNA, we compared the cumulative distribution functions (CDF) of the nuclear protein scores to that of the overall protein scores. One-tail Kolmogorov-Smirnov test was used to test if the two distributions are the same. With regard to the RNAs in the human MRP complex, Rnase P complex, and HOTAIR RNA, the p-values are 1.02E-16, 2.51E-125, and 2.18E-77, respectively. CDF images of the three lncRNAs are presented in Figure 2. We further collected the lncRNAs of Homo sapiens from the lncRNA database (http://www.lncrnadb.com), and deleted those sequences longer than 3000 due to the low computation speed of RNAsubopt software when dealing with long RNA sequences. Finally, 72 human lncRNAs were obtained. With the cutoff set at 0.05, 79.1% (57 out of 72) of these lncRNAs have significant p-values (Additional file 5: Table S4). Thus, most of these human lncRNAs we collected have significantly higher interaction scores with nuclear proteins.

Following a similar procedure, we also compared the CDF of the RNA-binding protein scores to that of the overall protein scores for each given lncRNA. Among the 20224 protein sequences of Homo sapiens we collected, 639 were annotated as the RNA-binding proteins according to the Cellular Component of Gene Ontology (GO:0003723, RNA binding, downloaded from BioMart, Additional file 4: Table S3). 56.9% (41 out of 72) sequences have significant p-values (Additional file 6: Table S5). Thus, more than half of these lncRNAs have significantly higher predicted interaction scores with known RNA-binding proteins.

## Conclusions

In this study, we introduced a new method lncPro for the prediction of protein associations with lncRNAs. Compared with existing methods, our method shortened the time for training matrix **M**. This matrix was also found to be theoretically optimal based on the model being used. Our method is computational friendly and does not lead to nonsensical cross terms. The comparison results with CatRAPID also show that our method has enhanced abilities of predicting the associations between lncRNAs and proteins. Specifically, we found the human lncRNAs we collected tend to interact with nuclear proteins and RNA-binding proteins.

However, the process of finding proteins that directly interact with a given lncRNA is still unsatisfactory because of the large number of proteins. Only when the complex is provided can the prediction of interaction within the complex be more accurate. Also, most RNA-protein pairs in our training set exist in the ribosome, so the training data might not be general enough. Though we tested the method on some non-ribosome complexes and it performed well. We should still be aware of the limited range of cases being used. To conduct a more accurate prediction, further work needs to be performed and more information should be considered. The use of Fisher’s linear discriminant method provides direction on how to incorporate different information into the prediction process.

Another thing is that lncPro meets some computational issues when dealing with very long non-coding RNAs. This is limited by the computational ability of RNA secondary structure prediction program. We will update lncPro when new software is available. When we study a long RNA sequence, sometimes we are only interested in certain sections of this sequence. lncPro can be applied if the particular section is not too long.

## Competing interests

The authors have declared that no competing interests exist.

## Authors’ contributions

QL carried out the bioinformatics analysis and drafted the manuscript. SR helped to carry out the bioinformatics analysis and drafted the manuscript. ML built the online server. YZ, DZ and XZ participated in its design and helped to draft the manuscript. TL conceived the study and participated in its design and coordination, and helped to draft the manuscript. All authors read and approved the final manuscript.

## Supplementary Material

Additional file 1: Table S1 The number of sequences contained in 44 complexes. Click here for file

Additional file 2: Table S2 The number of sequences in the remaining 18 complexes. Click here for file

Additional file 3: Figure S1 Distribution of Interaction Score. The distribution of predicted interaction scores for the shuffled set. The shuffled set was got by randomizing all pairs in the non-redundant negative training set. Click here for file

Additional file 4: Table S3 ID list of proteins. Click here for file

Additional file 5: Table S4 Test result of nuclear proteins. Click here for file

Additional file 6: Table S5 Test result of RNA-binding proteins. Click here for file
