# Supplementary material for: Computational prediction of associations between long non-coding RNAs and proteins
Source: BMC Genomics. 2013 Sep 24;14:651. doi: 10.1186/1471-2164-14-651 (PMC3827931; doi:10.1186/1471-2164-14-651)
Supplement: Additional file 3: Figure S1 — Distribution of Interaction Score. The distribution of predicted interaction scores for the shuffled set. The shuffled set was got by randomizing all pairs in the non-redundant negative training set. [file 1471-2164-14-651-S3.docx]

Distribution of Interaction Score

Figure S1 **Distribution of Interaction Score.** The distribution of predicted interaction scores for the shuffled set. The shuffled set was obtained by randomizing all pairs in the non-redundant negative training set.
